# Development of a Ten-lncRNA Signature Prognostic Model for Breast Cancer Survival: A Study with the TCGA Database

**DOI:** 10.1155/2020/6827057

**Published:** 2020-08-18

**Authors:** Wenqing Zhou, Yongkui Pang, Yunmin Yao, Huiying Qiao

**Affiliations:** ^1^Department of General surgery, The Fifth People's Hospital of Wujiang Area, Suzhou, Jiangsu 215211, China; ^2^Department of General practice, Suzhou Ninth People's Hospital, Suzhou, Jiangsu 215200, China

## Abstract

Long noncoding RNA (lncRNA) plays a critical role in the development of tumors. The aim of our study was construction of a lncRNA signature model to predict breast cancer (BRCA) patient survival. We downloaded RNA-seq data and relevant clinical information from the Cancer Genome Atlas (TCGA) database. Differentially expressed lncRNA were computed using the “edgeR” package and subjected to the univariate and multivariate Cox regression analysis. Corresponding protein-coding genes were used for Gene Ontology (GO) and Kyoto Encyclopedia of Genes and Genome (KEGG) pathway analysis. Finally, 521 differentially expression lncRNA were obtained. We constructed a ten-lncRNA signature model (LINC01208, RP5-1011O1.3, LINC01234, LINC00989, RP11-696F12.1, RP11-909N17.2, CTC-297N7.9, CTA-384D8.34, CTC-276P9.4, and MAPT-IT1) to predict BRCA patient survival using the multivariate Cox proportional hazard regression model. The C-index was 0.712, and AUC scores of training, test, and entire sets were 0.746, 0.717, and 0.732, respectively. Univariate Cox regression analysis indicated that age, tumor status, N status, M status, and risk score were significantly related to overall survival in patients with BRCA. Further, the multivariate analysis showed that risk score and M status had outstanding independent prognostic values, both with *p* < 0.001. The Gene Ontology (GO) function and KEEG pathway analysis was primarily enriched in immune response, receptor binding, external surface of plasma membrane, signal transduction, cytokine-cytokine receptor interaction, and cell adhesion molecules (CAMs). Finally, we constructed a ten-lncRNA signature model that can serve as an independent prognostic model to predict BRCA patient survival.

## 1. Introduction

Breast cancer (BRCA) is the most commonly diagnosed cancer and the leading cause of cancer death among women worldwide [1]. Although BRCA death is declining due to early detection and improved treatment, significant variability in patient outcomes remains. Almost 62667 died of this malignancy in 2018 [2]. The prognosis of BRCA is affected by multiple factors including age, tumor size, grade, lymph node involvement, histology, hormone-receptor status, HER-2 status, and positive margins [3]. Many clinical prediction models for predicting patient prognosis and disease-free survival have been proposed, mainly focusing on age at diagnosis; post-menopausal status; ER, HER-2, and ki-67 status; tumor size; lymph node involvement; metastasis; and therapeutic strategy [4–7]. These models are difficult to implement in clinical practice due to incomplete diagnostic characteristics and model limitations.

Next-generation sequencing (NGS) allows continuing identification of biomarkers for tumor diagnosis and prognosis. Such models increasingly focus on mRNA, miRNA, and lncRNA. Li et al. [8] used NGS data available in several databases, including the Geno Expression Omnibus (GEO), the Cancer Genome Atlas (TCGA), the Human Protein Atlas, and the International Cancer Genome Consortium, to construct a six-gene model (SQSTM1, AHSA1, VNN2, SMG5, SRXN1, and GLS) to assist clinicians in selecting personalized treatment for patients with hepatocellular carcinoma. Shi et al. [9], using the TCGA, identified a five-lncRNA signature model (AC069513.4, AC003092.1, CTC-205M6.2, RP11-507K2.3, and U91328.21) to inform the prognosis of patients with clear cell renal cell carcinoma. Lv et al. [10] constructed a six-mRNA signature model (TMEM252, PRB2, SMCO1, IVL, SMR3B, and COL9A3) that may aid prognosis for patients with triple-negative BRCA. Zhu et al. [11] built a four-lncRNA signature model (PVT1, MAPT-AS1, LINC00667, and LINC00938) to predict BRCA survival, but the AUC value for the time-dependent receiver operation characteristic (ROC) curve was only 0.64. To date, few studies of multi-lncRNA signatures in BRCA are available, and functions and mechanisms of lncRNA in BRCA have yet to be explored.

In this study, we deeply mined and analyzed high-throughput sequencing data and clinical characteristics from the TCGA. We subsequently developed a ten-lncRNA signature model that effectively predicts BRCA survival and demonstrated its independence from clinical factors.

## 2. Material and Methods

### 2.1. Data Source

The RNA-seq data and corresponding clinical information were mined from TCGA: http://cancergenome.nih.gov/). As of December 2019, 1164 clinical samples and relevant gene expression information were obtained. BACA samples with repeated or incomplete prognostic information were eliminated. Ultimately, 1035 BRCA samples and 111 normal samples were collected for the construction of the model and coexpression analysis. TCGA is an open public database and, ethics approval was not needed for the study.

### 2.2. Identification of Differentially Expressed lncRNA

RNA count data were obtained from the TCGA data portal, and expression levels of lncRNA and mRNA were determined using Reads Per Kilobase of exons per Million mapped Reads (RPKM). Potential lncRNA were identified based on (1) transcriptome sequences mapped in corresponding lncRNA rather than protein-coding regions, (2) transcriptome sequences annotated in GENCODE data, and (3) transcription sequences expressed in at least half of BRCA tissues. The expression profile of lncRNA was defined as mean RPKM ≥ 0.1 of 1146 BRCA samples. Finally, a total of 6129 lncRNA were enrolled. Differentially expressed lncRNA were identified using the R software “edgeR” package.

### 2.3. Statistical Analysis and Definition of the lncRNA-Related Prognostic Model

BRCA samples (1035 sequences) were randomly divided into a training set (*n* = 518) and a test set (*n* = 517). The Univariate Cox regression analysis was used to examine associations among lncRNA expression levels and overall survival (OS) in the training set. lncRNA were considered significant when *p* values were <0.05. These sequences were then used for the stepwise multivariate Cox regression analysis, using the R package “survival” (choose a model by AIC in a stepwise algorithm) [12–14]. Based on expression levels and coefficients (*β*) from multivariate Cox proportional hazards regression analysis, a novel ten-lncRNA-based prognostic risk score formula was defined [13–16]. The risk score formula was as follows:

(1) $$\begin{array}{c} \text{risk score}=\overset{N}{\underset{i=1}{\sum }}\text{E}\text{x}{\text{p}}_{i}\times \beta_{i}. \end{array}$$

A risk score for each patient in the training set was then calculated. BRCA patient samples can be divided into high-risk and low-risk groups, respectively, based on the median risk score as a cutoff. The Kaplan–Meier survival curve and the log-rank test were used to assess the prognostic value of the risk score using the R package “survival.”

The receiver operation characteristic (ROC) curve analysis within 3- and 5-year, using the R package “survivalROC,” compared sensitivity and specificity of survival predictions. Subsequently, we compared model predictions with traditional clinical risk factors (age, risk, stage, metastasis, tumor size, and lymph node involvement) using the univariate and multivariate Cox analysis. We also reassessed the relationship between risk level and clinical characteristics using the chi-square test. *p* < 0.05 was considered statistically significant. All data were analyzed using R scripts (version 3.6.1). All the figures were plotted by ggplot2 (version 3.2.1).

### 2.4. Functional Enrichment Analysis

To explore functional implications of lncRNA, Spearman's correlation coefficients were calculated between related lncRNA and protein-coding genes. Related protein-coding genes were screened for functional enrichment analysis. We subsequently performed GO analysis and the Kyoto Encyclopedia of Genes and Genome (KEGG) pathway enrichment analysis of differential expression protein-coding genes using the Database for Annotation, Visualization, and Integration Discovery (DAVID version 6.7).

## 3. Results

### 3.1. Differentially Expressed lncRNA and Clinical Characteristic

**Figure 1:**
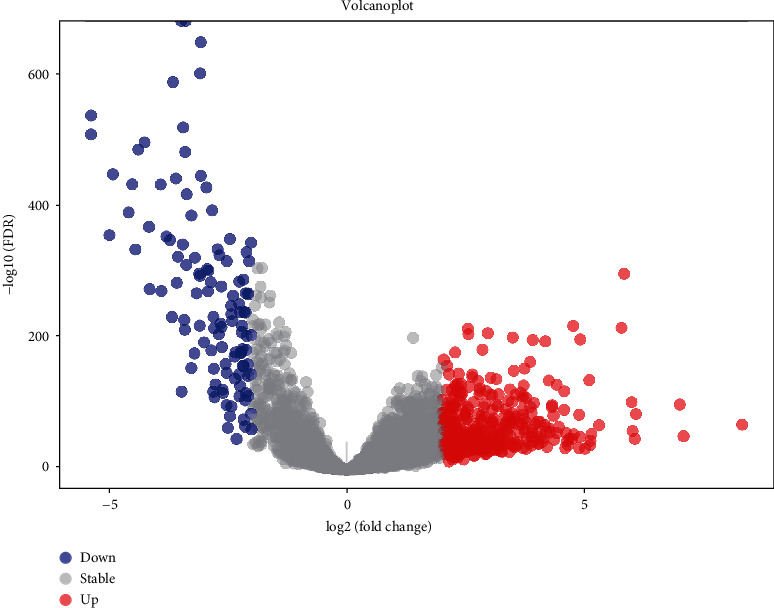
The volcano diagram of differentially expresses lncRNA between breast cancer tissue and normal tissue samples. Red dots represent upregulated lncRNA, and blue dots represent downregulated lncRNA. Cutoff for |log2 (fold change)| is 2, FDR < 0.001.

We analyzed specific baseline clinical characteristics of 1035 BRCA patients (Table 1). We selected lncRNA expression profiles from raw RNA-seq expression data, and then, differentially expressed lncRNA between BRCA samples and normal samples were identified following ∣log2 fold change (log2FC) | >2 and false discovery rate (FDR) < 0.001. This analysis recognized 406 upregulated lncRNA and 115 downregulated lncRNA (Figure 1).

### 3.2. Identification of lncRNA Associated with the OS of Patients from the Training Set and Validated in Test Set and Entire Sets

**Figure 2:**
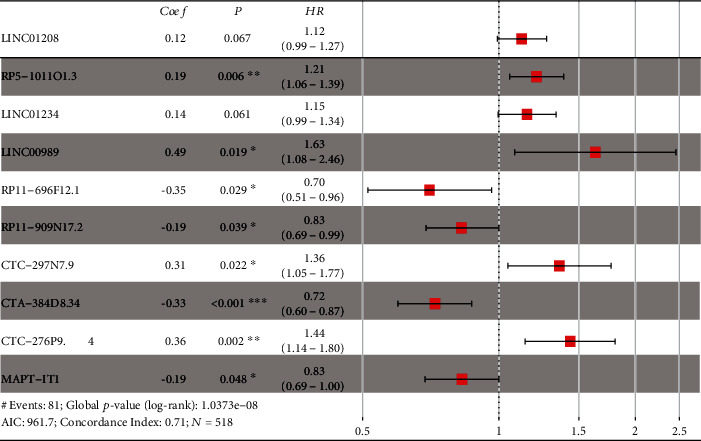
Forest plot of the ten-lncRNA signature risk model. Coef: coefficient; P: *p* value; HR: hazard ratio; *N*: samples in the training set; AIC: Akaike information criterion.

**Figure 3:**
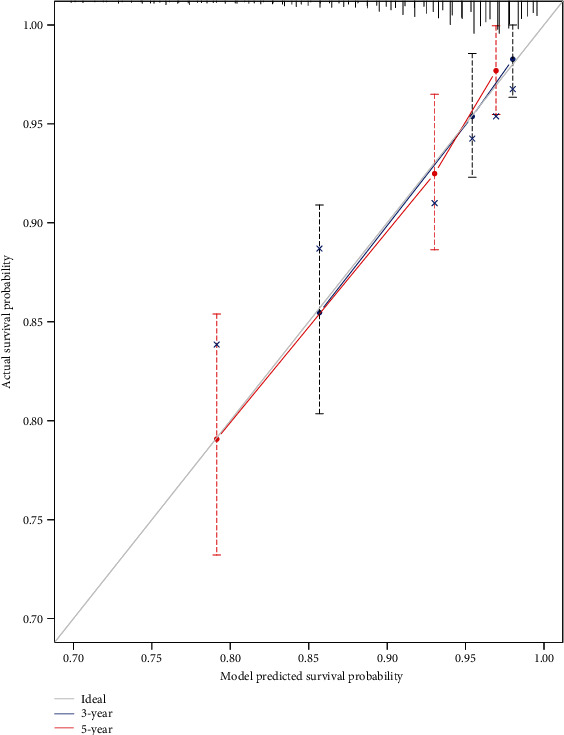
Calibration curve of the prognostic model. The gray line indicates the ideal plot for the calibration curve. 3-year: 3-year overall survival predicted probability by model. 5-year: 5-year overall survival predicted probability by model. The bootstrap method was performed.

The training set was first analyzed to identify possible prognostic lncRNA; then, the test and entire sets were used for validation. We performed univariate and multivariate Cox regression analysis to identify correlation among differentially expressed lncRNA and OS of BRCA patients using the training set. Finally, 32 of the 521 differentially expressed lncRNA were found to be associated with survival time (*p* < 0.05) by performing univariate Cox regression analysis (Table 2). In addition, a prognostic model, composed of 10 lncRNA, was established by performing a stepwise multivariate Cox proportional hazard regression model (Figure 2). risk score = (0.12 × expression level of^“^LINC01208^”^) + (0.19 × expression level of^“^RP5 − 1011O1.3^”^) + (0.14 × expression level of^“^LINC01234^”^) + (0.49 × expression level of^“^LINC00989^”^) + (−0.35 × expression level of^“^RP11 − 696F12.1^”^) + (−0.19 × expression level of^“^RP11 − 909N17.2^”^) + (0.31 × expression level of^“^CTC − 297N7.9^”^) + (−0.33 × expression level of^“^CTA − 384D8.34^”^) + (0.36 × expression level of^“^CTC − 276P9.4^”^) + (−0.19 × expression level of^“^MAPT − IT1^”^). The C-index for model was 0.712 (CI 0.686-0.740), and the calibration curve showed good performance for the prognostic model (Figure 3).

**Figure 4:**
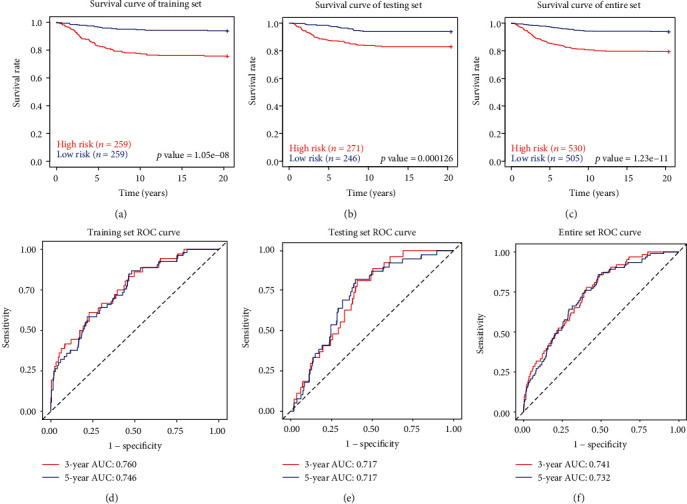
The ten-lncRNA-related risk score model predicts the OS of patients with BRCA in the training set, test set, and entire set. The Kaplan–Meier survival curves show a correlation between the expression of ten-lncRNA signature and overall survival of patients (a–c). ROC curves show the sensitivity and specificity of the ten-lncRNA signature in predicting 3- and 5-year survival rate (d–f). ROC curve: receiver operating characteristic curve. AUC: area under curve. These curves were performed by R package “survival,” “survivalROC,” and “ggplot2”.

Patients in the training set were classified into high-risk groups and low-risk groups using median risk score (0.938) as a cutoff. The survival rate of the high-risk group was significantly lower than the low-risk group in the Kaplan–Meier method and the log-rank test (Figure 4(a)). Subsequently, the prognostic ability of the model was assessed by calculating AUC of the time-dependent ROC curve. Generally, an AUC from 0.7 to 0.9 is deemed reliable. The AUC value of the training set was 0.760 in 3 years, 0.746 in 5 years (Figure 4(d)), indicating that the ten-lncRNA signature model shows high sensitivity and specificity.

We next validated our ten-lncRNA signature model with test and entire data sets. We again used risk scores for all patients in test and entire sets and divided patients into high- and low-risk groups based on the same threshold from the training set. Again, we found that survival rate of the high-risk group was significantly lower than the low-risk group in both test and entire sets. (Figures 4(b) and 4(c)). Time-dependent ROC curve analysis for the ten-lncRNA signature model achieved an AUC score of 0.717 in both 3- and 5-year for the test set, and the AUC score of the entire set was 0.741 in 3 years and 0.732 in 5 years, respectively (Figures 4(e) and 4(f)).

### 3.3. Independence of the Ten-lncRNA Signature and Other Clinical Variables

We assessed whether survival prediction based on the 10-lncRNA signature model was independent of clinical characteristics. Univariate Cox regression analysis indicated that age, tumor status, N status, M status, and risk score were significantly related to OS in the entire set. Also, the multivariate Cox analysis indicated that risk score and M status show outstanding independent prognostic value, both with *p* < 0.001 (Table 3). Further, based on *χ*^2^ tests, the risk level had no association with age, stage, metastasis, tumor size, and lymph node involvement (Table 4). Collectively, our study demonstrates that the ten-lncRNA signature prognostic model is a robust tool for predicting the prognosis of BRCA patients.

### 3.4. Functional Characteristic of Ten Prognostic lncRNA

Biological functions of lncRNA remain unclear, but the expression of lncRNA is remarkably correlated with neighboring protein-coding genes. We obtained expression profiles of protein-coding genes from raw RNA-seq data and extracted corresponding protein-coding genes with ten lncRNA. Spearman's correlation coefficients with ∣COR | >0.5 and *p* < 0.05 as the cutoff yielded 1178 protein-coding genes for stepwise functional enrichment analysis.

**Figure 5:**
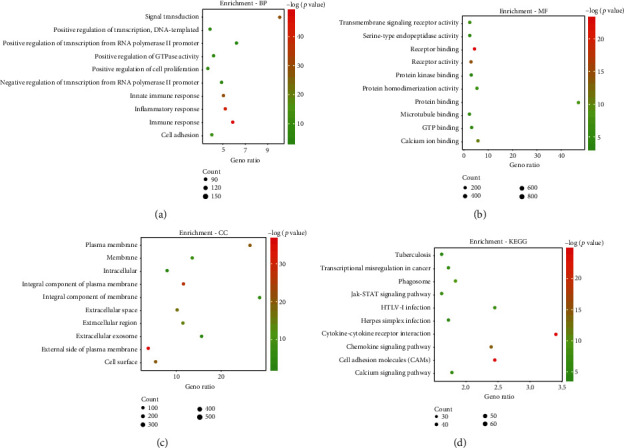
Top 10 potential biological functions of the protein-coding genes associated with ten lncRNA according to *p* < 0.05. (a) BP: biological process. (b) MF: molecular function. (c) CC: cellular component. (d) KEGG: Kyoto Encyclopedia of Genes and Genomes.

The GO function and KEGG pathway enrichment analysis of protein-coding genes used DAVID bioinformatics resources 6.7. BP results showed that protein-coding genes were enriched for signal transduction, immune response, inflammatory response, and positive regulation of transcription from RNA polymerase II promoter (Figure 5(a)). Characteristics of enrichment in MF were primarily transmembrane signaling receptor activity, protein binding, protein homodimerization activity, calcium ion binding, and receptor binding (Figure 5(b)). For CC analysis, genes were enriched in integral components of the plasma membrane, integral components of membranes, plasma membrane, extracellular exosome, and external side of the plasma membrane (Figure 5(c)). Also, results from the KEGG pathway analysis were enriched for cytokine-cytokine receptor interaction, cell adhesion molecules (CAMs), calcium signaling pathway, transcriptional dysregulation in cancer, and HTLV-I infection (Figure 5(d)).

## 4. Discussion

High-throughput sequencing technology produces increasing amounts of sequencing data for studies of cancer diagnosis, therapy, and prognosis [17]. Current studies focus on ncRNA associated with cancer, especially lncRNA. Some studies confirm that lncRNA plays a crucial role in th4e occurrence and progress of tumors, such as gastric [18], colon [19], and BRCAs [20].

In the present study, we downloaded RNA-seq data and clinically relevant information related to BRCA from the TCGA database. We obtained 1164 clinical samples and corresponding gene expression information. A total of 521 differently expression lncRNA involved in BRCA were pulled from the TCGA database, including 406 upregulated and 115 downregulated genes. Subsequently, univariate and multivariate Cox regression analyses identified correlations among differentially expressed lncRNA and OS in a training set. These correlations were used to establish a risk model for predicting BRCA prognosis. A 10-lncRNA signature risk prediction model (LINC01208, RP5-1011O1.3, LINC01234, LINC00989, RP11-696F12.1, RP11-909N17.2, CTC-297N7.9, CTA-384D8.34, CTC-276P9.4, and MAPT-IT1) was produced. Patients were subdivided into high- and low-risk groups based on the median risk score. Three-year AUC values for the time-dependent ROC curve in the training, test, and entire sets were 0.760, 0.717, and 0.741, respectively, indicating outstanding performance in survival prediction. Recently, Zhu et al. [11] and Li et al. [21] have proposed a breast cancer prognosis model based on RNA-seq, and three-year AUC values of their models are 0.641 and 0.711 in the training set, respectively. Therefore, the performance of our model outperforms these two models. We also compared the risk model and clinical parameters (age, stage, metastasis, tumor size, and lymph node involvement) using univariate and multivariate Cox analysis. The prognostic value of the model was independent of other clinical factors in BRCA, but the functional relationship between risk score and tumor development was unclear.

Previously, Liao et al. [22] showed that LINC01234 knockdown suppressed cell proliferation, migration, and invasion of colorectal cancer cells, while blocking the cell cycle and inducing cell apoptosis. Chen et al. [23] found that LINC01234 functioned as a ceRNA for miR-304-5p, resulting in derepression of its endogenous target core-binding factor. In addition, Chen et al. [24] found that LINC01234 expression is increased in non-small-cell lung cancer tissues, and its upregulation is associated with metastasis and shorter survival. Downregulation of LINC01234 impairs cell migration and invasion *in vitro* and inhibits cell metastasis *in vivo* by serving as a competing endogenous RNA for the miR-340-5p and miR-27b-3p; LINC01234 also affects RNA-binding proteins LSD1 and EZH2, leading to histone modification and transcriptional repression of antiproliferative gene BTG2 [24, 25]. In another study, Wang et al. [12] found that the expression of RP11-909N17.2 is positively associated with colorectal cancer outcomes and prognosis; in our study, RP11-909N17.2 is a protective factor in BRCA prognosis. This discrepancy requires further study.

LINC00989 and MAPI-IT1 are associated with congenital diseases [26, 27], and their relationship with cancer remains unclear. No studies have reported associations between LINC01208, RP5-1-11O1.3, RP11-696F12.1, CTC-297N7.9, CTA-384D8.34, CTC-276P9.4, and cancer, but we speculate that these lncRNA may be involved in BRCA tumorigenesis. More research effort is necessary to test this hypothesis.

Many issues remain to be addressed. First, we only download data from the TCGA database. More data are available in other databased that could prove valuable for the risk model. Second, lncRNA play important roles in the occurrence and progress of tumors, but the function of lncRNA in the signature is unclear. Additional experimental study of these lncRNA may help understand functional mechanisms and thus the functional basis for the ten lncRNA for prognosis of BRCA.

## 5. Conclusions

We identified differentially expressed gene associated with the pathogenesis of breast cancer and constructed a ten lncRNA prognostic model to predict prognosis of patients with BRCA. The prognostic model presented a good performance in 3- and 5-year OS prediction. Functional mechanisms of these lncRNA have not yet been investigated. Prospective studies are needed to further validate the utility of the ten-lncRNA prognostic model.

## Figures and Tables

**Table 1:** Demographic characteristics of 1035 breast cancer patients.

Characteristic	Training	Test	Entire
	Set (*n* = 518)	Set (*n* = 517)	Set (*n* = 1035)
Age (years)			
Mean	58.3	58.13	58.21
<50	143	144	287
≥50	375	373	748
Clinical stage			
I	86	87	173
II	307	291	598
III	113	127	240
IV	12	12	24
T stage			
T1	136	133	269
T2	304	298	602
T3	62	66	128
T4	16	20	36
N stage			
N0	237	247	484
N1	185	166	351
N2	67	56	123
N3	29	48	77
M stage			
M0	506	505	1011
M1	12	12	24
Vital status			
Living	437	454	891
Dead	81	63	144
OS time (years)			
Mean	17.99	18.52	18.26
Range	0-20.42	0-20.42	0-20.42

Age: the age of patient at diagnosis; OS time: overall survival time.

**Table 2:** 32 survival-related lncRNA were obtained based on univariate Cox regression (*p* < 0.05).

Name	Coef	HR	*p* value	Name	Coef	HR	*p* value
ST8SIA6-AS1	0.09034361	1.0945503	0.026243	RP11-909N17.2	-0.20319	0.81612	0.019443
LINC01208	0.12624424	1.1345592	0.034391	RP11-320N21.2	0.237757	1.268401	0.018486
RP11-127O4.3	0.11482258	1.1216744	0.027422	MAFA-AS1	-0.26894	0.764193	0.006172
RP11-148B18.3	-0.1436104	0.8662251	0.028363	LA16c-325D7.1	-0.18608	0.830209	0.043436
RP11-95M15.1	0.13719873	1.1470561	0.041031	CTC-297N7.9	0.254558	1.289891	0.049002
LINC01105	0.14482779	1.1558405	0.009837	MAPT-AS1	-0.10879	0.896916	0.033228
RP11-644C3.1	0.18463926	1.2027845	0.009652	LINC00668	0.125025	1.133177	0.009984
AC011294.3	0.19606627	1.2166075	0.042138	RP5-1028K7.2	-0.21427	0.80713	0.021088
AC061961.2	-0.1268231	0.8808895	0.018243	RP11-806H10.4	-0.27203	0.761831	0.005325
RP5-1011O1.3	0.1777658	1.1945455	0.004379	RP11-127I20.5	-0.20671	0.813254	0.04301
RP11-320N21.1	0.20684929	1.2297972	0.035183	RP3-395M20.12	-0.23466	0.79084	0.008974
RP11-386B13.4	0.1560986	1.1689415	0.047225	CTA-384D8.34	-0.25528	0.774697	0.005498
LINC01234	0.16039376	1.173973	0.021267	RP13-49I15.6	-0.2677	0.765138	0.037547
BMPR1B-AS1	-0.1032851	0.9018698	0.030654	CTD-3001H11.2	-0.23828	0.787981	0.034354
LINC00989	0.45410227	1.574759	0.023186	CTC-276P9.4	0.260139	1.29711	0.017559
RP11-696F12.1	-0.3033194	0.7383632	0.043388	MAPT-IT1	-0.19551	0.822418	0.03155

HR: hazard ratio. Coef: coefficient.

**Table 3:** Univariate and multivariate Cox regression analyses in the training, test, and entire set.

Variables		Univariable analysis			Multivariable analysis	
	HR	95% CI of HR	*p* value	HR	95% CI of HR	*p* value
Training set (*n* = 518)						
Age	1.021	1.004-1.038	0.014	1.016	0.999-1.034	0.055
T stage	1.574	1.183-2.094	0.002	1.166	0.812-1.674	0.405
N stage	1.588	1.275-1.979	<0.001	1.269	0.893-1.803	0.183
M stage	8.277	4.132-16.582	<0.001	3.647	1.354-9.825	0.011
Stage	2.202	1.616-2.999	<0.001	1.099	0.585-2.063	0.769
Ten-lncRNA risk score	4.203	2.462-7.176	<0.001	3.775	2.202-6.471	<0.001
Testing set (*n* = 517)						
Age	1.013	0.994-1.032	0.170			
T stage	1.312	0.947-1.819	0.103			
N stage	1.337	1.062-1.682	0.013	1.113	0.758-1.635	0.585
M stage	12.332	6.231-24.408	<0.001	11.382	3.605-35.933	<0.001
Stage	2.068	1.452-2.946	<0.001	1.078	0.568-2.048	0.818
Ten-lncRNA risk score	2.885	1.636-5.088	<0.001	3.196	1.803-5.668	<0.001
Entire set (*n* = 1035)						
Age	1.018	1.005-1.030	0.006	1.017	1.005-1.030	0.007
T stage	1.445	1.166-1.791	<0.001	1.068	0.823-1.385	0.620
N stage	1.445	1.235-1.691	<0.001	1.152	0.894-1.483	0.274
M stage	10.125	6.233-16.450	<0.001	6.202	2.882-13.349	<0.001
Stage	2.1347	1.691-2.696	<0.001	1.168	0.727-1.876	0.522
Ten-lncRNA risk score	3.5227	2.388-5.196	<0.001	3.396	2.298-5.018	<0.001

HR: hazard ratio. CI: confidence interval.

**Table 4:** The relationship between clinical parameters and risk score.

Subgroup	Training set			Test set			Entire set		
	High risk	Low risk	*p* value	High risk	Low risk	*p* value	High risk	Low risk	*p* value
Age (years)			0.492			0.626			0.407
<50	68	75		73	71		141	146	
≥50	191	184		198	175		389	359	
T stage			0.71			0.901			0.481
T1	65	71		68	65		133	136	
T2	154	150		157	141		311	291	
T3	30	32		34	32		64	64	
T4	10	6		12	8		22	14	
N stage			0.547			0.819			0.547
N0	111	126		129	118		240	244	
N1	95	90		84	82		179	172	
N2	37	30		30	26		67	56	
N3	16	13		28	20		44	33	
M stage			0.243			0.678			0.263
M0	251	255		264	241		515	496	
M1	8	4		7	5		15	9	
Clinical stage			0.378			0.868			0.525
I	40	46		43	44		83	90	
II	149	158		156	135		305	293	
III	62	51		65	62		127	113	
IV	8	4		7	5		15	9	
Vital status			<0.001			<0.001			<0.001
Living	195	242		224	230		419	472	
Dead	64	17		47	16		111	33	

Chi-square test was used.

## Data Availability

The raw data and relevant R code used to support the findings of this study are recorded in https://github.com/zhouwenqing789/TCGA-MODEL-LCNRNA-BRCA.git
